# Induced lineage promiscuity undermines the efficiency of all-trans-retinoid-acid-induced differentiation of acute myeloid leukemia

**DOI:** 10.1016/j.isci.2021.102410

**Published:** 2021-04-11

**Authors:** Yijia Tang, Xin Tian, Zihan Xu, Junke Cai, Han Liu, Nan Liu, Zhu Chen, Saijuan Chen, Feng Liu

**Affiliations:** 1 Shanghai Institute of Hematology, State Key Laboratory of Medical Genomics, National Research Center for Translational Medicine at Shanghai, Ruijin Hospital Affiliated to Shanghai Jiao Tong University School of Medicine, Shanghai 200025, China; 2 Interdisciplinary Research Center on Biology and Chemistry, Shanghai Institute of Organic Chemistry, Shanghai 200032, China

**Keywords:** Cancer, Transcriptomics

## Abstract

All-trans retinoid acid (ATRA) can induce terminal differentiation of acute promyelocytic leukemia (APL), also known as the M3 subtype of acute myeloid leukemia (AML). However, non-APL types of AML respond poorly to ATRA-induced differentiation, and the mechanism underlying cell-type-specific resistance against ATRA remains unclear. Here, we use single-cell transcriptome analysis to compare the differentiation trajectories of two AML cell types during ATRA treatment. We show that in NB4 (APL/AML-M3) cells, ATRA activates canonical myeloid lineage factors—including SPI1, CEBPE, and STAT1—to direct near-normal differentiation toward mature granulocytes. By contrast, in HL60 (AML-M2) cells, ATRA-induced differentiation is incomplete and promiscuous, which is characterized by coinduction of both myelopoiesis and lymphopoiesis gene expression programs, as well as transient activation of cis-regulatory elements associated with myeloid differentiation. Our study suggests that the differentiation inducing capacity of ATRA in certain subtypes of AML may be compromised by therapy-induced lineage promiscuity.

## Highlights

• Single-cell analysis of ATRA-induced differentiation in two AML cell types
• In AML-M3/APL cells, ATRA induces a near normal trajectory of granulopoiesis
• In AML-M2 cells, ATRA induces incomplete differentiation and lineage promiscuity
• ATRA-induced lineage promiscuity involves transient cis-regulatory reprogramming

## Graphical abstract

**Figure:**
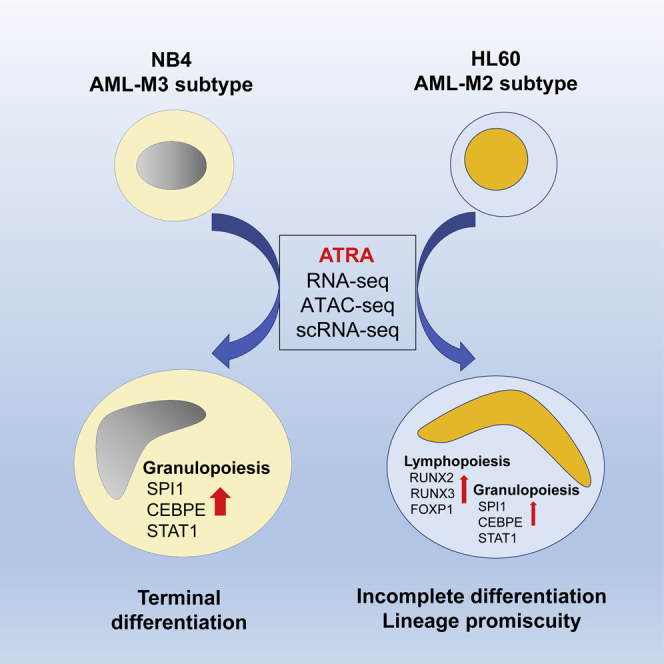

## Introduction

The hematopoietic system is generated by a hierarchy of stem/progenitor cells in the bone marrow, which proliferate and differentiate along distinct cell lineages, leading to terminal differentiation of a multitude of specialized cell types of the myeloid compartment (e.g. granulocytes, monocytes, macrophages, erythrocytes, mast cells, and dendritic cells) and the lymphoid compartment (e.g. B cells, T cells, and natural killer cells) (Laurenti and Gottgens, 2018). It has been suggested that leukemia often originates from aberrant differentiation blockage of hematopoietic progenitor cells, and unblocking such blockage may provide an efficient means of cure (Tenen, 2003). Indeed, this strategy, known as differentiation therapy, has been successfully applied in the treatment of acute promyelocytic leukemia (APL), also known as acute myeloid leukemia (AML)-M3 subtype, using all-trans retinoid acid (ATRA) in combination with arsenic compounds (Chen and Chen, 2017; Huang et al., 1988; Petrie et al., 2009; Wang and Chen, 2008). Earlier studies have suggested that ATRA has the potential to induce differentiation in non-APL types of AML as well as other cancers such as embryonic carcinoma, breast cancer, and neuroblastoma (de The, 2018; Petrie et al., 2009; Thiele et al., 1985). However, non-APL cancers generally respond poorly to ATRA-based differentiation therapy and the mechanisms underlying such cancer-specific resistance remain obscure.

ATRA belongs to the retinoid family of signaling molecules (Tang and Gudas, 2011). Activation of the retinoid signaling pathway is mediated by converting a transcriptional complex, composed of heterodimers of nuclear retinoid (RARα, β and γ) and rexinoid (RXRα, β and γ) receptors, from transcription repressor to transcription activator (Altucci et al., 2007). In APL, t(15; 17) chromosomal translocation produces the PML-RARA fusion protein, which prevents the RA signaling pathway from being activated at physiological levels of retinoids (de The, 2018; Kakizuka et al., 1991; Tang and Gudas, 2011). A large body of evidence has shown that the transregulatory suppressor function of PML-RARA can be relieved by pharmacological levels of ATRA, leading to restoration of a normal granulocyte differentiation program and loss of clonal expansion capacity of APL-initiating cells (de The, 2018; Jiao et al., 2013) (Tan et al., 2020). In non-APL types of AML, a lack of therapeutic effect of ATRA is generally thought to be the lack of PML-RARA mutation, although, in principle, terminal differentiation programs may still be activated by ATRA via the intrinsic retinoid signaling pathway. However, the differentiation-inducing capacity of ATRA in most cancer cell types has not been well characterized, in part, owing to the difficulty in relating the developmental trajectory of tumor cells to those of their closely related cell types.

Here, we attempted to address the mechanisms of AML subtype-specific responses to ATRA using two well-established promyelocyte cell lines. The first cell line, NB4, was derived from a patient with APL with t(15; 17) chromosomal translocation. It expresses the PML-RARA fusion protein and can be induced by ATRA to terminally differentiate into granulocyte-like cells (Lanotte et al., 1991). The other line, HL60 (HL-60), was initially reported to derive from a patient diagnosed with APL (Collins et al., 1978) but was later re-defined as an AML-M2-like case owing to the absence of t(15; 17) translocation and its molecular profile featuring amplification of the Myc oncogene (Dalton et al., 1988). *In vitro*, HL60 can also be induced by ATRA to differentiate to granulocyte-like cells (Ramirez et al., 2017). However, a study using cDNA microarray experiments found few ATRA-regulated genes shared between NB4 and HL60 cells (Zheng et al., 2005), suggesting that very different gene expression programs were induced by ATRA in each cell type, although how such differences reflect ATRA-induced differentiation programs has not been explored.

In this study, we have applied single-cell RNA-seq analysis to delineate the trajectories of ATRA-induced differentiation in NB4 and HL60 cells, reasoning that a comparison of cell-type-specific differentiation processes may provide insights into the variations of ATRA's therapeutic efficacy between APL and non-APL types of AML. Our analysis indicates that in NB4, ATRA induces a near-normal process of terminal granulopoiesis. But, in HL60, ATRA-induced differentiation is incomplete and promiscuous, showing coactivation of granulopoiesis and lymphopoiesis gene expression programs. Through single-cell gene expression and gene network analyses, we also uncovered the complete sets of transcription factors underlying ATRA-induced differentiation. Furthermore, by profiling ATRA-regulated cis-regulatory elements during induced differentiation, we found that incomplete differentiation in HL60 cells is accompanied by weak and transient reprogramming of a group of cis-regulatory elements associated with myelopoiesis. We discuss the implication of these findings in understanding the efficiency of ATRA-based differentiation therapy in different subtypes of AML.

## Results

### Overview of ATRA-regulated gene expression programs in NB4 and HL60 leukemia cell lines

**Figure 1:**
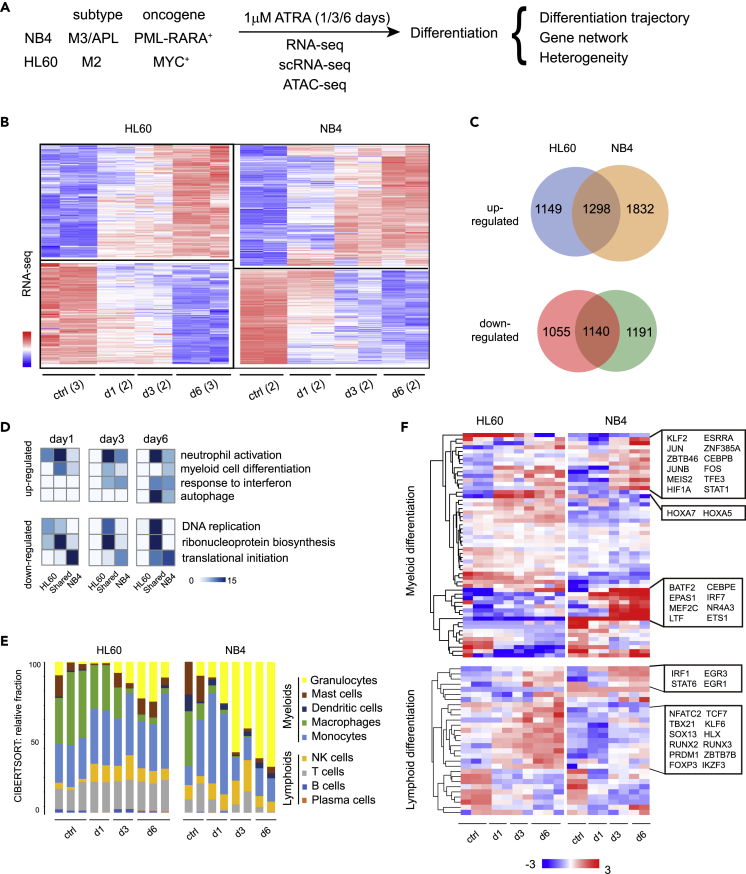
Overview of cell type-specific responses of leukemia cells to ATRA (A) Experimental scheme. NB4 and HL60 cells were incubated with 1 μM of ATRA in culture and collected at different time points for bulk and single cell RNA-seq analysis. (B) Heat map of DEGs in the RNA-seq data using HL60 and NB4 cells treated with 1 μM of ATRA for 1/3/6 days. Ctrl: control/untreated; day 1/3/6: day 1/3/6 after treatment. Numbers of biological repeats were shown in brackets. (C) Venn diagrams of shared and cell type-specific DEGs between cell lines. (D) Heat map of enrichment scores (-Log_10_p value) of select GO terms related to ATRA-regulated biological processes. (E) CIBERSORT analysis of the RNA-seq data. The relative fraction of the transcriptome signatures of major immune cell types is shown. (F) Heatmap of TFs associated with myeloid and lymphoid differentiation in the RNA-seq data. Select TF clusters genes are indicated on the right.

To systematically compare ATRA-regulated gene expression programs between HL60 and NB4 cells, we first performed a side-by-side mRNA-seq experiment using each cell line cultured with 1 μM of ATRA for 1/3/6 days (Figure 1A). As compared with untreated controls, 5461 and 4642 differentially expressed genes (DEGs) were identified in HL60 and NB4 cells after ATRA treatment (Wald test, padj <0.05), respectively. Most DEGs changed in a progressive manner, with 2438 genes shared between cell lines (1298 were upregulated; 1140 were downregulated) (Figures 1B and 1C, see also Tables S1 and S2). Gene ontology (GO) analysis indicated that the shared, upregulated DEGs were significantly associated with myeloid differentiation, neutrophil activation, and interferon response; the shared, downregulated DEGs were significantly associated with processes such as the cell cycle and metabolism (Figure 1D). Furthermore, 1832 activated DEGs private to NB4 showed enrichment of genes related to myeloid development and maturation (e.g. interferon response and autophagy) during the course of ATRA treatment, whereas 1149 activated DEGs private to HL60 only showed enrichment in myeloid activation genes at day 1 after treatment (Figure 1D). These studies indicated that even though NB4 and HL60 cells can both be induced to differentiate toward mature hematopoietic cell types, there were significant differences in the gene expression programs induced in each cell type, with ATRA-induced NB4 cell expressing more genes associated with the myeloid lineage.

To assess how these transcriptome profiles correlated with differentiation, we used the CIBERSORT algorithm to compute the relative similarity of each transcriptome to those a panel of major immune cell types (Newman et al., 2015). The transcriptome signature of granulocytes was most strongly and consistently increased in ATRA-treated NB4 cells (p value <0.05, one-way ANOVA), but its change in ATRA-treated HL60 cells was not as significant. By contrast, the signature of NK cells showed notable, albeit weak, increases in ATRA-treated HL60 cells (p value <0.05, one-way ANOVA), but such change was less obvious in ATRA-treated NB4 cells (Figure 1E). In addition, we retrieved genes associated with myeloid and lymphoid differentiation from the GO database and found that most of them showed ATRA-dependent upregulation in a cell-type-specific manner. In particular, a group of myeloid-differentiation genes (e.g. CEBPE, BATF2, MEF2C) were activated to high levels in NB4 but not in HL60. In addition, a group of lymphocyte-differentiation TFs showed coordinated increase in ATRA-induced HL60 cells (e.g. NFATC2, RUNX2, RUNX3), in consistence with weak increase of lymphocyte-related transcriptome signatures (Figure 1F; see also Figures S1 and S2, Table S3).

Together, these results not only confirmed that ATRA induced highly cell-type-specific gene expression programs in NB4 and HL60 cells but also revealed a certain degree of heterogeneity of the gene regulatory networks activated in both cell types. Nevertheless, in these bulk RNA-seq experiments, a pool of cells were used as input, and the results only provided averaged gene expression profiles, which were insufficient to resolve the fate of individual cells or potential heterogeneity in their differentiation trajectories (Kolodziejczyk et al., 2015). Therefore, in subsequent experiments, we turned to Drop-seq (Macosko et al., 2015), a high-throughput droplet-based scRNA-seq platform, to investigate the trajectories of ATRA-induced differentiation in each cell type at single-cell levels.

### Single-cell analysis of ATRA-induced NB4 cells

**Figure 2:**
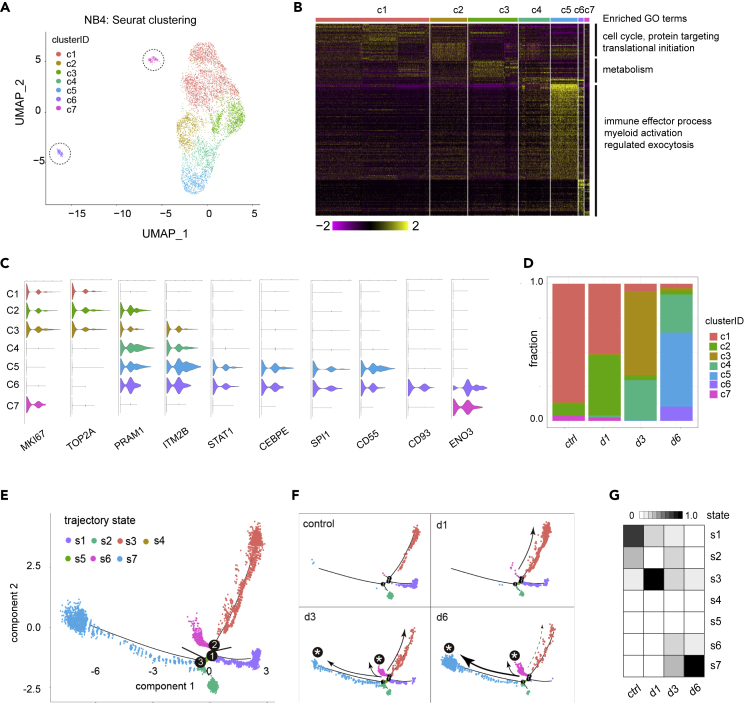
Single-cell analysis of ATRA-induced differentiation of NB4 (A) Seurat clustering scRNA-seq experiments using 6,135 NB4 cells with or without ATRA treatment for up to 6 days (1,673 control/untreated cells, 2,068 ATRA day 1 cells, 1,261 ATRA day 3 cells, and 1,133 ATRA day 6 cells). The majority of cells were closely positioned in U-MAP. Two separate clusters are circled. (B) Heat map of marker genes in different clusters. Enriched GO terms for different groups of genes are shown at right. (C) Violin plots of select genes showing cluster specificity. (D) Fraction of cells in different clusters per experiment. (E) Pseudotime trajectory constructed by monocle revealed seven states and three branch points. (F) Distribution of cells derived from different experiments in the trajectory tree. (G) The fraction of cells in different states per experiment.

We started our single-cell analysis with ATRA-induced NB4 cells, with the aim of using this PML-RARA^+^ APL cell type as reference for studying ATRA-induced differentiation trajectory. To do so, we collected NB4 cells cultured with 1 μM of ATRA treatment for 1/3/6 days (untreated cells were used as controls) and performed Drop-seq experiments in replicates for each time point. A strict quality control pipeline was used to remove doublets and low-quality cells (see transparent methods for details). In total, this data set consisted of 6,135 single-cell transcriptomes (1,673 control/untreated cells; 2,068 ATRA day 1 cells; 1,261 ATRA day 3 cells; 1,133 ATRA day 6 cells). An unsupervised clustering analysis using this data set divided the cells into 7 clusters; of which, five (c1–c5) were close to each other, whereas the other two (c6 and c7) were separated (Figure 2A). Based on gene expression profiles and the duration of exposure to ATRA, the first six clusters (c1–c6) could be placed in a spectrum of differentiation toward granulocytes (Figures 2B–2D; see also Figure S3, Table S4). On one side of the spectrum were cells of c1, most of which were untreated, control cells, and marked by cell cycle genes (e.g. MKI67, a G1/S phase marker; TOP2A, an M phase marker), suggesting that they were proliferating naive NB4 cells (Figure 2C). On the other side of the spectrum were cells of c6, which resembled differentiated granulocyte-like cells because they were mainly composed of cells treated with ATRA for 6 days and marked by a large number of granulocyte genes (Figure 2B). For the intermediate clusters, c2 and c3 were marked by cell cycle genes and PRAM1, a known direct target of retinoid acid signaling (Moog-Lutz et al., 2001), suggesting that they had been exposed to ATRA but were yet to exit the cell cycle. Two other intermediate clusters, c4 and c5, were marked by many granulocyte differentiation-related genes, including three TFs—SPI1, CEBPE, and STAT1—that were known for directing granulocyte differentiation (Lekstrom-Himes et al., 1999; Nerlov and Graf, 1998; Wu et al., 2020). The levels of these genes showed a gradual increase from c4 to c5 and c6 (Figure 2B), which was indicative of a gradual process of ATRA-induced differentiation.

On the other hand, the cells in another separate cluster (c7) also expressed granulocyte-marker-like ENO3, which might suggest them as a distinct granulocyte-like cell type (Figures 2A–2C). However, c7 cells were mainly from untreated (control) experiment. In addition, they still expressed high levels of MKI67 but not granulopoiesis genes such as STAT1, CEBPE, and SPI1. Together, these features suggested that c7 represented a group of naive NB4 cells that spontaneously expressed a small set of granulocyte genes.

Next, we applied pseudotime analysis (Cacchiarelli et al., 2018) to infer ATRA-induced differentiation trajectory of NB4 cells. The resulted trajectory contained seven leaves (cellular states) connected by three branch points (Figure 2E). Projecting the sample collection time to this trajectory revealed that control cells resided in three states (s1–s3), with the majority in s1. Cells from day 1 were mainly in the same states as the control sample, yet the proportion of cells in s3 was greatly expanded (Figures 2F and 2G). In day 3 and day 6 samples, the fraction of cells in s3 progressively decreased, with a concomitant increase in s6 and then s7 (Figures 2F and 2G). These observations indicated s1 as the starting/root state and s7, the induced differentiated state. Furthermore, they suggested that the observed differentiation process was nonlinear, with multiple branches/states (i.e. s2, s3, and s6) transiently activated during the course of ATRA treatment.

### Single-cell analysis of ATRA-induced HL60 cells

**Figure 3:**
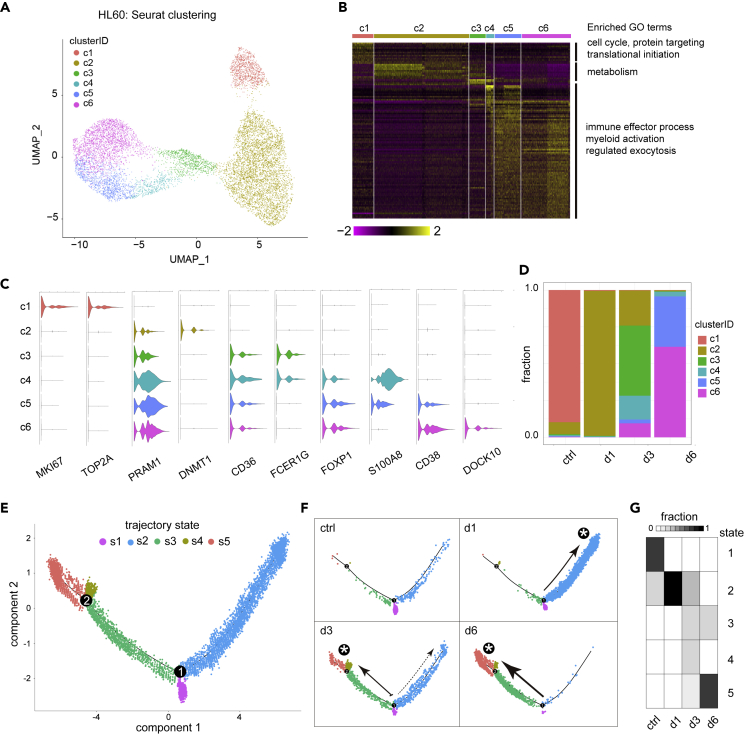
Single-cell analysis of ATRA-induced differentiation of HL60 (A) Seurat clustering scRNA-seq experiments using 14,389 cells HL60 cells with or without ATRA treatment for up to 6 days (2,118 control cells, 6,639 ATRA day 1 cells, 1,708 ATRA day 3 cells, and 3,924 ATRA day 6 cells). Expression based classification of cell clusters are labeled with colors. (B) Heat map of marker genes in different clusters. Enriched GO terms for different groups of genes are shown at right. (C) Violin plot of select genes showing cluster specificity. (D) Fraction of cells in different clusters per experiment. (E) Pseudotime trajectory constructed by monocle revealed five states and two branch points. (F) Distribution of cells derived from different experiments in the trajectory tree. (G) Heat map of the fraction of cells in different states per experiment.

We then applied the same scRNA-seq analysis pipeline on ATRA-induced HL60 cells. This data set included 10,041 cells in total (1,075 control cells; 4,007 ATRA day 1 cells; 1,495 ATRA day 3 cells; 3,464 ATRA day 6 cells). Gene expression profile-based clustering divided them into six clusters that could also be placed in a spectrum of differentiation (Figures 3A–3D; see also Figure S4, Table S4). Cells of c1 were mainly composed of naive HL60 cells from the control sample and marked by the expression of cell cycle genes such as MKI67 and TOP2A. Three intermediate clusters (c2–c4) were mostly cells treated with ATRA for 1 and/or 3 days. The gene expression profiles of c2 cells were similar to those of c1, representing ATRA-treated cells (PRAM1^+^) that were yet to start differentiation; c3 and c4 were marked by increased expression of myeloid differentiation genes such as CD36, FCER1G, and S100A8. The last two clusters (c5 and c6) were largely composed of cells treated with ATRA for 6 days and expressed higher levels of differentiation-related genes (Figure 3C). Of note, three granulocyte differentiation TFs—CEBPE, SPI1, STAT1—marking ATRA-induced NB4 cells were not detectable in any group of ATRA-induced HL60 cell types; instead, FOXP1 was found to be expressed at high levels in differentiated cell types (i.e. those in c4–c6) (Figure 3C).

Pseudotime-based differentiation trajectory of ATRA-treated HL60 cells was composed of five cellular states connected by two branch points (Figure 3E). Untreated HL60 cells resided in three states (s1–s3), with the majority in s1 (Figures 3F and 3G). The fraction of cells in state s2 was expanded in the day 1 but then gradually decreased in day 3 and day 6. At the same time, there was an increase of cell fractions in states s3, s4, and s5. By day 6, most cells were in s5 and a minority in s3 (Figures 3F and 3G). These observations thus indicated s2 and s4 as intermediate states and s3–s5 as differentiated states.

### Cell-specific differentiation trajectories induced by ATRA

**Figure 4:**
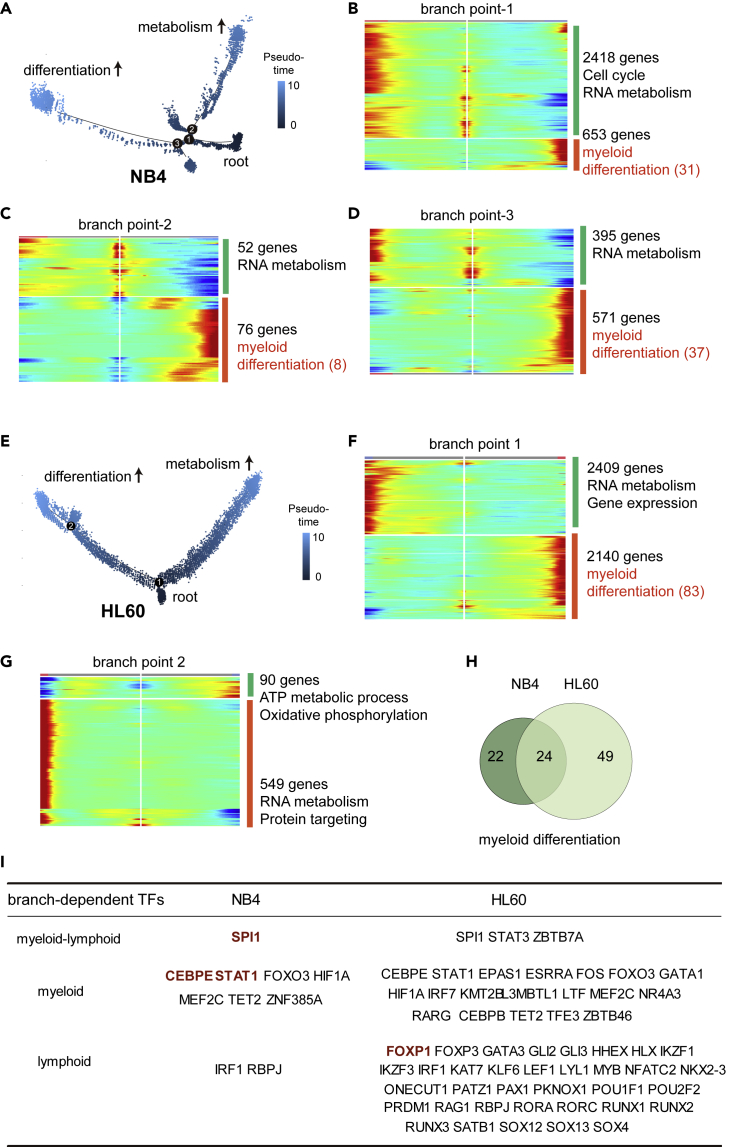
Comparison of ATRA-regulated gene sets during the differentiation of NB4 and HL60 (A) ATRA-induced NB4 differentiation trajectory as in Figure 2A. Cells were colored by pseudotime values. The root state and major branches with distinct gene expression profile changes are indicated. (B–D) Heat map of genes differentially expressed at each branchpoint of the NB4 trajectory tree. Representative GO-enriched biological process terms are shown on the right. The number of genes in each category was indicated. Genes related to myeloid differentiation or activation are colored in red. The numbers of genes in each group are shown. (E) ATRA-induced HL60 differentiation trajectory as in Figure 3A. Cells were colored by pseudotime values. The root state and two major branches with distinct gene expression profile changes are indicated. (F and G) Heat map of genes differentially expressed at each branchpoint of the HL60 trajectory tree. Representative GO-enriched biological process terms are shown on the right. The number of genes in each category was indicated. Genes related to myeloid differentiation or activation are colored in red. The numbers of genes in each group are shown. (H) Venn diagram of the number of myeloid differentiation genes in ATRA-induced NB4 and HL60 cells. (I) Table of branch-dependent TFs in ATRA-induced NB4 and HL60 cells.

We now return to comparing the differentiation trajectories of ATRA-induced NB4 and HL60 cells using the aforementioned single-cell data sets. Because multiple intermediates states appeared during induced differentiation of both NB4 and HL60, we performed branch-dependent gene expression analysis (Cacchiarelli et al., 2018) to determine if the same or different sets of genes were involved in cell fate decisions between them (Figures 4A–4G; see also Table S5).

In the NB4 trajectory, 3071, 128, and 966 branch-dependent genes were identified at branch points 1, 2, and 3, respectively. At each point, one set of genes was significantly associated with myeloid development, whereas another set, activated toward an alternative state, was associated with metabolic processes such as ribosome biogenesis and RNA processing. Furthermore, at branch point 1, which is close to the root state (i.e. s1), 31 myeloid differentiation genes were found (Figure 4B). Moving along the trajectory to branch point 2 toward the intermediate states s3 and s6, only 8 myeloid differentiation genes (8 of 31, 25.8%) were retrained (Figure 4C). By contrast, moving along the trajectory to branch point 3 toward the differentiated state (s7), most myeloid differentiation genes (21 of 31, 71.0%) were still present, and other myeloid-related genes were induced (Figure 4D). These observations indicated that a differentiation program was continuously activated by ATRA along the path toward differentiated states but was lost when the cells entered the intermediate states.

In the HL60 trajectory, 4549 and 639 branch-dependent genes were identified at branch points 1 and 2, respectively. At branch point 1, one set of branch-dependent genes was associated with myeloid development (83 myeloid differentiation genes and 164 myeloid activation genes) and the other set was associated with metabolic activities including ribosome biogenesis and RNA processing. At branch point 2, however, none of the branch-dependent gene sets were significantly associated with myeloid development; instead, both were enriched with genes involved in RNA metabolism and mitochondria activities (Figures 4E–4G). Thus, while a myeloid differentiation program is initiated as the cells left the root state and progressed toward some differentiating states at the first branch point, this program appeared to be discontinued at the second branch point.

Although many branch-dependent genes in both NB4 and HL60 were found to be associated with hematopoietic cell differentiation, only a few of them were shared (Figure 4H). Notably, the branch-dependent TFs in NB4 were predominantly associated with granulopoiesis, but only about one-half of the branch-dependent TFs in HL60 were associated with granulopoiesis, whereas the other half were associated with lymphopoiesis (Figure 4I). These results confirmed the coexpression of myelopoiesis and lymphopoiesis TFs, including RUNX2 and RUNX3, observed in the bulk RNA-seq analysis shown previously (compare Figures 4I and 1F).

### Cell-type-specific expression of lineage factors during ATRA-induced differentiation

**Figure 5:**
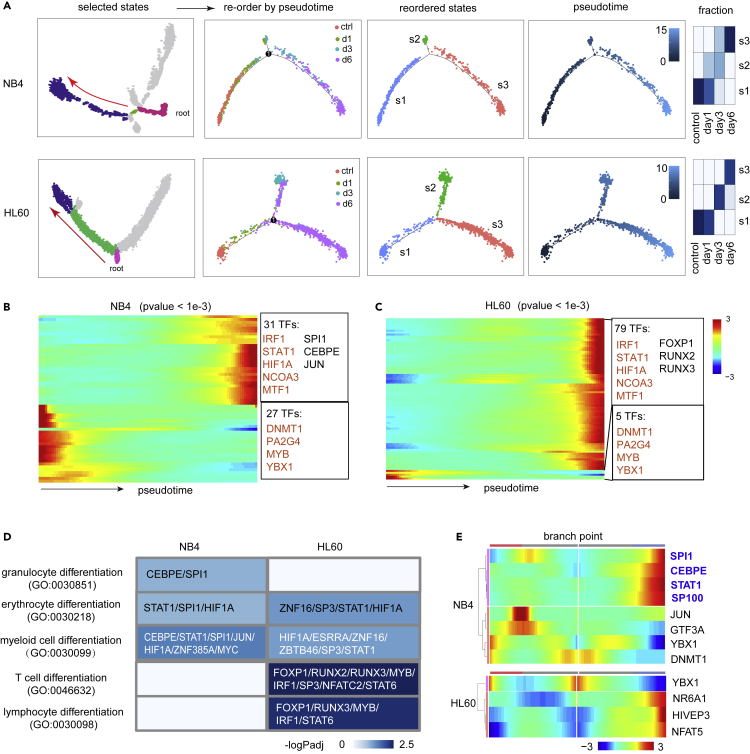
Comparison of the differentiation trajectories of ATRA-induced NB4 and HL60 (A) Cellular states along the differentiation path of NB4 and HL60 cells were selected and reordered by pseudotime. The resulted trajectories were colored by experiments, cellular states, and pseudotime values. Relative fraction of cells from different experiment in each state is shown on the right. (B and C) Heat map of pseudotime-dependent expression of TFs in NB4 (B) and HL60 (C) (p value <0.001). Select TFs are shown at right. Common branch-dependent TFs are colored in red. (D) Heat map of GO terms related to myeloid and lymphoid differentiation. Pseudotime-dependent TFs of each GO term are shown. (E) Heat map of branch-dependent TFs at the single branch points of the differentiation trajectories.

The single-cell differentiation trajectories of both NB4 and HL60 cells contained multiple differentiating/differentiated states and intermediate metabolically active states. We reasoned that those aligned along the differentiation axis might be used for a more focused analysis of ATRA-induced differentiation program. Therefore, we selected cells from each data set three states that constituted the shortest path from control cells to ATRA-induced differentiating/differentiated cell types and reordered them by pseudotime values (Figure 5A). The resulted trajectory of each cell type was a three-leaf structure containing a single branch point in which most cells were aligned along the major axis, and their pseudotime-time values were largely in accordance with the duration of ATRA treatment (Figure 5A).

Next, we retrieved all TF genes showing pseudotime-dependent expression patterns in each subtrajectory (p value <0.001, Figures 5B and 5C). Of them, 31 and 27 TFs were activated and repressed in NB4, respectively; 79 and 5 TFs were activated and repressed in HL60, respectively. Only 9 TFs were shared (five were activated: IRF1, HIF1A, STAT1, NCOA3, and MTF1; four were repressed: DNMT1, MYB, PA2G4, and YBX1) (Figures 5B and 5C). The pseudotime-dependent genes in NB4 were only significantly associated with myeloid lineages, whereas those in HL60 were significantly associated with both myeloid and lymphocyte lineages (Figure 5D). Furthermore, at the single branch point in the NB4 subtrajectory, canonical granulocyte differentiation TFs such as SPI1, CEBPE, and STAT1 were again found to be upregulated along the axis of differentiation (Figure 5E). However, in the branch point of HL60, the branch-dependent TFs were YBX1, NR6A1, HIVEP3, and NFAT5. Of them, only NFAT5 was previously implicated in T cell development (Trama et al., 2000). Thus, although many TFs associated myeloid and lymphocyte lineages were induced in HL60, they appeared not to be involved in the fate decision at a late phase of differentiation (i.e. after 3 days post ATRA treatment).

### Cell-type-specific gene regulatory networks induced by ATRA

**Figure 6:**
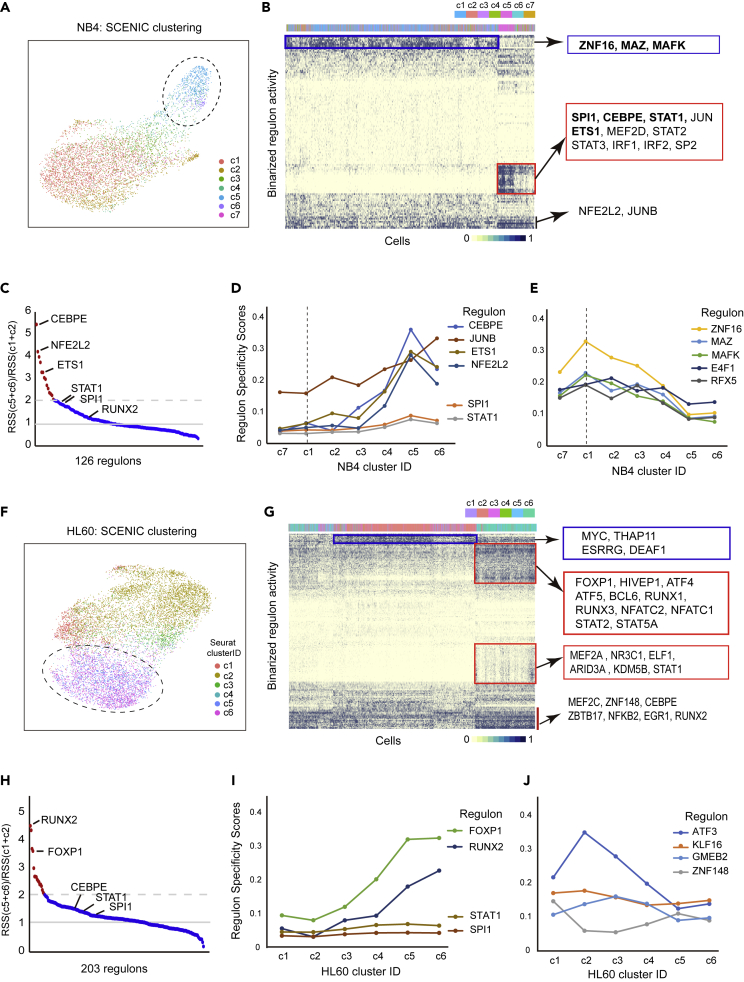
Single-cell gene regulatory network analysis of ATRA-induced NB4 and HL60 cells (A) UMAP plot using the SCENIC regulonAUC scores of NB4 scRNA-seq data. Cells are colored by their Seurat cluster ID as in Figure 2A. Circle indicates clustered differentiated cell types (c5 and c6). (B) Heatmap of binarized regulonAUC scores of SCENIC analysis of NB4 scRNA-seq data. X axis are cells; y axis are regulons (named by TF regulators). (C) Dot-plot of fold changes of RSS of 126 regulons in differentiated (c5 and c6) versus undifferentiated (c1 and c2) clusters of NB4. (D and E) Plots of regulon specificity scores (RSS) of selected regulons in each NB4 Seurat cluster. (F) UMAP plot using the SCENIC regulonAUC scores of HL60 scRNA-seq data. Cells are colored by their Seurat cluster ID as in Figure 3A. Circle indicates clustered differentiated cell types (c5 and c6). (G) Heatmap of binarized regulonAUC scores of SCENIC analysis of HL60 scRNA-seq data. X axis are cells; y axis are regulons (named by TF regulators). (H) Plot of fold changes of RSS of 203 regulons in differentiated (c5 and c6) vs. undifferentiated (c1 and c2) clusters of HL60. (I and J) Plots of regulon specificity scores (RSS) of selected regulons in each HL60 Seurat cluster.

To further determine how gene regulatory networks were regulated by ATRA, we used the SCENIC (Single-Cell rEgulatory Network Inference and Clustering) algorithm to compute the activities of coregulated TF-target gene sets (regulons) in the scRNA-seq data (Aibar et al., 2017). In both NB4 and HL60, the differentiated cell clusters annotated by gene expression profiles were grouped together by the regulon specificity scores (RSS), consistent with the expectation that TFs are critical regulators of cell differentiation (Figures 6A, 6B, 6F, and 6G; see also Figures S5 and S6, Table S6).

Examination of top ATRA-induced regulons in both NB4 and HL60 cells indicated that those highly increased in differentiating NB4 cells were primarily involved in the myeloid lineage (e.g. regulons of CEBPE, JUNB, ETS1, and NFE2L2; Figure 6D), whereas those highly increased in differentiating HL60 cells were primarily involved in the lymphoid lineage (e.g. regulons of RUNX2 and FOXP1; Figure 6I). Although the activities of many regulons were increased in both cell types, their induction levels notably differ. For example, in NB4, the top-ranked regulon was that of CEBPE, whose RSS increased by more than 5 times after ATRA induction, whereas its increase in HL60 cells was relatively small (Figures 6C and 6H). The converse pattern was observed for the RUNX2 regulon, which is the most highly increased in the HL60 data set, but its increase in NB4 is relatively small (Figures 6C and 6H). On the other hand, the activities of a number of regulons were decreased after ATRA-induced differentiation in both cell lines, which may reflect the loss of gene regulatory activities associated with the identity of parental cell lines (Figures 6E and 6J).

### Dynamic changes of chromatin accessibility in response to ATRA

**Figure 7:**
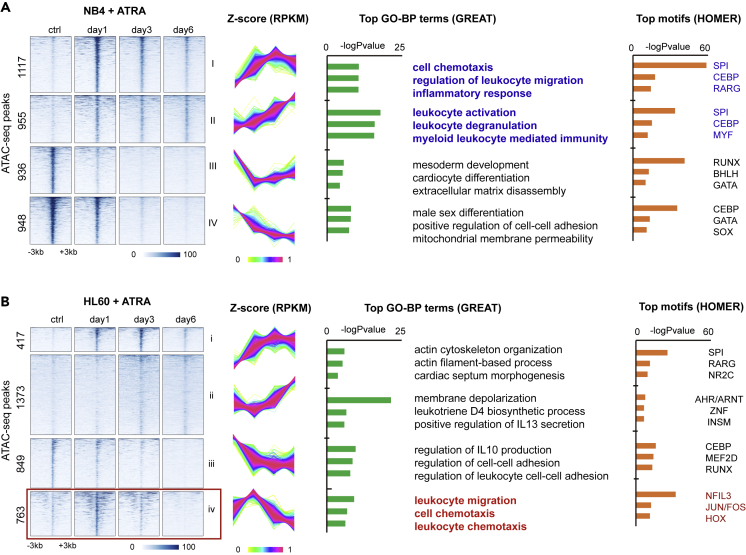
ATRA-regulated accessible chromatin during induced differentiation of NB4 and HL60 (A) ATAC-seq experiments of ATRA-treated NB4 cells. Dynamically changed accessible chromatin regions (ACRs) are shown, which are divided into four clusters by k-means clustering (k = 4). The heatmap of normalized peak intensity, plot of z-scores, top associated GO Biological Process (GO-BP) terms, and top enriched TF-binding motifs are shown from left to right. ACRs of groups I and II are associated with GO terms and TF related to myeloid development and leukocyte migration (color in blue). (B) ATAC-seq experiments of ATRA-treated HL60 cells. Dynamically changed ACRs are also divided into four clusters by k-means clustering (k = 4). The heatmap of normalized peak intensity, plot of z-scores, top associated GO Biological Process (GO-BP) terms, and top enriched TF-binding DNA motifs are shown from left to right. ACRs of group iv (highlighted by red rectangle) are transiently activated during ATRA treatment and are associated with GO terms and TFs related to leukocyte migration (color in red).

Finaly, as an independent assay of ATRA-regulated transcriptional programs during differentiation of NB4 and HL60 cells, we performed the assay for transposase-accessible chromatin using sequencing (ATAC-seq) to profile the landscape of chromatin accessibility of each cell line cultured with 1 μM of ATRA for 1/3/6 days. This assay uses the Tn5 transposase to preferentially digest the chromatin regions with low-histone-binding activities, allowing for identification of accessible chromatin regions (ACRs) enriched with active cis-regulatory elements such as enhancers and promoters (Buenrostro et al., 2015). Using these data, we identified 3956 and 3402 ATAC-seq peaks showing dynamic changes over time in NB4 and HL60, respectively (Figures 7A and 7B, see also Table S7).

To explore the dynamic patterns of these ACRs, we used an unsupervised clustering analysis to divided them into four groups (Figures 7A and 7B). In NB4, the accessibility about half of the dynamical ACRs increased (groups I and II), whereas that of the other half decreased (groups III and IV), during the course of ATRA treatment (Figure 7A). Genomic Regions Enrichment of Annotations Tool analysis indicated that the activated ACRs, but not silenced ones, were significantly associated with genes related to leukocyte activation, degranulation, migration, and inflammatory response; in addition, the DNA sequences of these ACRs were enriched with binding sties for the SPI and CEBP protein families (Figure 7A). Taking into consideration of the induction of SPI1 and CEBPE in ATRA-induced NB4 cells, these results are indicative of cis-regulatory reprogramming of granulocyte differentiation driven by these two canonical granulopoiesis factors. By contrast, the overall change of chromatin accessibility in ATRA-regulated ACRs in HL60 is relatively weaker (Figure 7B). Although the accessibility of 1790 ACRs (groups i and ii) showed consistently increase during the course of ATRA-treatment, they were not significantly associated with myeloid development, which is in spite of significant enrichment of DNA binding motifs for the SPI protein family. Moreover, the accessibility of one group (group iv) in HL60 were transiently increased at day 1 after the treatment but then decreased to the baseline level. Interestingly, these transiently activated ACRs were associated with genes related to leukocyte migration and chemotaxis (Figure 7B). These results are in agreement with the aforementioned transcriptome analyses that ATRA did not induce a complete myelopoiesis program in HL60 cells.

## Discussion

Here, we have compared the differentiation trajectories of two AML model cell lines that represented the APL/AML-M3-subtype (i.e. NB4) and AML-M2-subtype (i.e. HL60) respectively. We find that in NB4, ATRA induces three key granulopoiesis factors—SPI1, CEBPE, and STAT1—to direct a differentiation trajectory resembling the process of terminal granulopoiesis. By contrast, in HL60, the granulopoiesis program is only weakly induced; at the same time, a lymphopoiesis program—primarily regulated by RUNX2, RUNX3, and FOXP1—is activated, making the differentiation trajectory of ATRA-induced HL60 cells appear incomplete and promiscuous. The distinction between these differentiation trajectories is mainly delineated by expression- and pseudotime-based single-cell transcriptome analyses, using samples collected at discrete time points during the course of ATRA treatment. Although such analyses are subjected to influences from sampling time and the number of cells obtained from each time point, the very different modes of ATRA-induced differentiation processes are also supported by bulk transcriptome and cis-regulatory landscape analyses in this study.

The seeming activation of a lymphopoiesis program in HL60 is somewhat unexpected, considering that it is a promyelocyte cell line derived from a patient with AML (Collins et al., 1978; Dalton et al., 1988). Nevertheless, most, perhaps all, signal transduction pathways are pleiotropic in terms of regulating different sets of target genes in different cellular context (Barolo and Posakony, 2002). For example, in early development of the hematopoietic system, the retinoid signaling pathway has been shown to be involved in the control of stem cell fate in addition to that of myeloid differentiation (Tang and Gudas, 2011). Thus, the retinoid pathway may have the potential to control some lymphopoiesis genes, which are normally silent but then inappropriately activated by ATRA in HL60. On the other hand, despite significant upregulation of many lymphopoiesis-related TFs, it appears that ATRA-induced HL60 cells did not undergo terminal differentiation along either the myeloid or lymphoid lineage. Instead, they seemed to be stalled at a cell fate decision point at about 3 days after ATRA treatment, whereby both canonical myelocyte- and lymphocyte-determining TFs ceased to driving further differentiation. The mechanism underlying the dissociation between these TFs and terminal differentiation in ATRA-treated HL60 cells is unclear, though one possibility is that the accessible chromatin landscape in HL60 may only favor myelopoiesis. This possibility is consistent with the observation that the dynamically changed accessible chromatin regions in both HL60 and NB4 are primarily enriched with binding motifs for myelopoiesis-TFs, but not lymphopoiesis-TFs.

Broadly speaking, the coinduction of lymphopoiesis and myelopoiesis programs in ATRA-induced HL60 cells could be referred to as a type of lineage promiscuity. Previously, the phenomenon of lineage promiscuity has been reported during early stage of the hematopoiesis, whereby some multipotent progenitors coexpress low levels of lineage-determining factors responsible for multiple cell types (e.g. myeloids and lymphoids) (Greaves et al., 1986; Miyamoto and Akashi, 2005). Developmental lineage promiscuity, however, is often transient and disappears when the progenitor cells become committed toward differentiation into a given cell type (Laiosa et al., 2006). On the other hand, long-lasting lineage promiscuity has been shown in certain cancer cells, manifested as constitutive expression of markers of different cell types as a result of genetic mutations disrupting factors of gene regulation (Greaves et al., 1986). By contrast, the observed lineage promiscuity in HL60s is actively induced by ATRA. Therefore, it represents a type of therapy-induced lineage promiscuity, which becomes evident only after exposure to external stimulus (e.g. ATRA).

Our single-cell analysis also illustrated several layers of nuance in ATRA-induced cell differentiation. In the trajectories of both NB4 and HL60, there is at least one branch point bifurcating into two paths: one leads to differentiated cell states; the other leads to intermediate states showing downregulation of differentiation genes and upregulation of RNA processing and ribosome biogenesis genes, which might indicate a form of metabolically active state. Such metabolically active states appeared to be occupied by most cells treated by ATRA within the first three days (NB4 and HL60 alike), whereas differentiating/differentiated states become evident only after more than three days of continuous ATRA treatment. At present, it is not yet known whether cells on the path to differentiation originate from the high-metabolic cells or a small fraction of cells at earlier times that did not enter the high metabolic state(s). Furthermore, after six days of ATRA treatment, a small percent of NB4 cells appeared to remain undifferentiated (31 of 1133 ATRA day 6 cells in c1, 2.73%, Figure 2D). It is unclear whether these cells represented a group of rare ATRA-resistant cells that existed in the original cell culture or reflected intrinsic heterogeneous response to a given treatment through epigenetic reprogramming. Further studies using single-cell lineage tracing technologies may shed light on these issues (VanHorn and Morris, 2021).

In summary, our study uncovered two heretofore underappreciated side effects of ATRA's pleiotropic effects. First, it can activate a large number of genes related to ribosome biogenesis that do not appear to be directly linked with induced cell differentiation. Second, ATRA may simultaneously activate both myelopoiesis and lymphopoiesis programs in certain AML cells (i.e. HL60), leading to a mixed and incomplete differentiation phenotype. We suggest that induced lineage promiscuity may serve as a model to describe the lack of efficacy of ATRA-based differentiation therapy in non-APL cancer cells (e.g. HL60-like AML-M2 cells), and methods to restrict promiscuous gene expression programs may help enhance the efficiency of ATRA-dependent differentiation therapy in non-APL type of leukemia.

### Limitations of the study

This study used two AML cell lines to investigate the trajectories of ATRA-induced differentiation. While NB4 cells has been shown by numerous studies to mimic the response of APL to ATRA-based differentiation therapy, it is not clear at present whether HL60 cells recapitulate the response of AML-M2 cells in patients *in vivo*. This is mainly because ATRA has not been approved for treating non-APL types of AML. Therefore, whether tumor cells from AML-M2 patients also show incomplete and lineage promiscuity remains to be verified. In future studies, this issue may be addressed by experiments using patient-derived leukemia cells cultured with ATRA *in vitro*. Furthermore, this study is performed using isolated cell cultures, which does not address potential influence of tumor cell microenvironment on the therapeutic effect of ATRA. Finally, the HL60 cell line has been commonly referred to as the AML-M2 subtype, based on the earlier morphology-based French-American-British classification system (Dalton et al., 1988). However, the current WHO classification system tentatively includes more than 20 AML subtypes, each characterized by distinct mutation profiles and pathophysiological features (Arber et al., 2016). The main genetic mutations of HL60 are known (MYC amplified; CDKN2A, NRAS, and TP53 mutated) (Dalton et al., 1988), but owing to significant heterogeneity in the mutation profiles between patients with AML and subtypes, it is still unclear which of the WHO-classified AML subtype HL60 might represent. Thus, cautions shall be taken in extrapolating this study to the interpretation of clinical responses of different AML subtypes to ATRA-based differentiation therapy.

### Resource availability

#### Lead contact

Feng Liu (lf12034@rjh.com.cn)

#### Materials availability

No new or unique reagents were generated or used in this study.

#### Data and code availability

The next-generation sequencing data generated in this study are publicly available from The National Omics Data Encyclopedia (NODE; https://www.biosino.org/node) with the accession number OEP001921. NGS data analyses are performed using open-source tools as described in the transparent methods section in supplemental information.

## Methods

All methods can be found in the accompanying Transparent Methods supplemental file.

## Supplemental information

Document S1. Transparent methods and Figures S1–S6

Table S1. Summary of differentially expressed genes of NB4 RNA-seq data, related to figure 1

Table S2. Summary of differentially expressed genes of HL60 RNA-seq data, related to figure 1

Table S3. Lists of genes related to myeloid or lymphoid differentiation, related to figure 1

Table S4. Summary of marker genes of scRNA-seq clusters, related to figures 2 and 3

Table S5. Summary of pseudotime analysis of scRNA-seq data, related to figure 4

Table S6. Summary of SCENIC analysis of scRNA-seq data, related to figure 6

Table S7. Summary of time course analysis of ATAC-seq data, related to figure 7

## Footnotes

Supplemental information can be found online at https://doi.org/10.1016/j.isci.2021.102410.
