# Supplementary material for: Induced lineage promiscuity undermines the efficiency of all-trans-retinoid-acid-induced differentiation of acute myeloid leukemia
Source: iScience. 2021 Apr 11;24(5):102410. doi: 10.1016/j.isci.2021.102410 (PMC8099557; doi:10.1016/j.isci.2021.102410)
Supplement: Document S1. Transparent methods and Figures S1–S6 [file mmc1.pdf]

iScience, Volume *24*

Supplemental information

Induced lineage promiscuity undermines the efficiency of all-trans-retinoid-acid-induced differentiation of acute myeloid leukemia

Yijia Tang, Xin Tian, Zihan Xu, Junke Cai, Han Liu, Nan Liu, Zhu Chen, Saijuan Chen, and Feng Liu

Transparent Methods

Cell culture

NB4 cells were gift from M. Lanotte (Institut National de la Santé et de la Recherche Médicale, Paris) (Lanotte et al., 1991). HL60 (HL-60) cells were kindly provided by the Stem Cell Bank, Chinese Academy of Sciences. The identity of each cell line was independently authenticated by short tandem repeat (STR) DNA profiling and querying against the ATCC STR Database (Genewiz, Inc). Both cell lines were cultured in Iscove's Modified Dulbecco's Medium (IMDM) supplemented with 10% fetal bovine serum (MOREGATE), 100 units/mL penicillin and 100 mg/mL streptomycin (GIBCO) at 37 °C with 5% CO_2. ATRA was purchased from SelleckChem (Cat# S1653) and dissolved in DMSO as a stock solution at 1 mM. For cell differentiation induction experiment, the ATRA stock was diluted to 1 μM in cell culture media for indicated incubation time.

RNA-Seq

Total RNA was extracted from approximately 1 million cells. 1-2 μg of RNA per sample was used for sequencing library preparation using VAHTS Total RNA-seq Library Prep Kit for Illumina (Cat# NR601, Vazyme) following the manufacturer’s recommendations. Multiplexed libraries were mixed and sequenced by Illumina Xten at the depth of 50-60 million 150 bp pair-end reads. The sequencing reads of each library were pseudoaligned to a transcriptome index file (based on Homo_sapiens.GRCh38.cdna.all.fa) using Kallisto (v0.46.0) (Bray et al., 2016). The resulted transcript compatibility counts were used for differential gene expression analyses using the DESeq2 R package (Love et al., 2014). CIBERSORT analysis was performed by uploading the gene expression matrix to the online tool server (https://cibersort.stanford.edu/), and LM22 signature gene file and 100 permutations were chosen for the analysis (Newman et al., 2015). The results of DEG analysis are summarized in Tables S1-S2. Genes associated with human myeloid and lymphoid differentiation are assembled from GO database (www.geneontology.org) and listed in Table S3.

Drop-seq

Single cell RNA-seq was performed using Drop-seq Library Protocol version 3.1 (12/28/15) according to Macosko et. al. (Macosko et al., 2015). Two biological replicates were performed for each experiment (control, ATRA_1d, ATRA_3d, and ATRA_6d) using NB4 or HL60 cells. Each library was sequenced in a flowcell lane by Illumina Xten using a 150bp paired-end sequencing kit with a custom read1 primer (Macosko et al., 2015). After sequencing, reads were filtered and sorted by their barcodes of origin and aligned to the reference transcriptome (GRCh38.gencode.v25.gene.annotation) using Drop-seq core computational protocol version 2.3.0 (https://github.com/broadinstitute/Drop-seq/releases). Mapped reads were quantified into UMI-filtered counts per gene. The resulted digital gene expression (DGE) matrices were imported to R programming language environment for clustering and pseudotime analyses.

Single cell transcriptome analysis

ScRNA-seq data analysis was performed as follows:

1) The DGE matrix of each experiment was imported to the Seurat R package (v3.1.5) (Butler et al., 2018) to filter out cells of low quality and doublets. Only cells expressing at least 200 genes and only genes that were expressed in at least 0.1% of all cells per sample were retained. Cells with more than 20% mitochondrial genes then removed. SCTransform was used to regress out variations of mitochondria gene expression, followed by principal component analysis using the RunPCA function. Based on the PCElbowPlot result, 8-12 principal components (PCs) were selected to identify clusters. Putative doublets were identified by the DoubletFinder (v2.0.2) R package assuming 10% doublet formation rate, after which highly expressed genes in each cluster were identified by the FindAllMarkers function (Wilcoxon rank sum test, only.pos = TRUE, logfc.threshold = 0.1). For each cluster, if the top 5 marker genes are mitochondria genes, then cluster was deemed as apoptotic and discarded. The DGE matrices containing the remaining cells were saved for subsequent data analysis.

2) the DGE matrices of each cell line (control, ATRA-day1, ATRA-day3, ATRA-day6) were merged and imported into Seurat for SCTransform and graph-based clustering as above. Clusters with less than 1% of all cells in the dataset were discarded, and the remaining cells were subject to another round of SCTransform and graph-based clustering until all clusters contain more than 1% of all cells. These filtering and re-clustering steps were repeated once or twice to empirically remove putative poor quality cells that compose small and diffusive clusters without distinct cell type-specific markers. After the final round of cell filtering, marker genes for each cluster were identified by FindAllMarkers function (Wilcoxon rank sum test, only.pos = TRUE, logfc.threshold = 0.2). Clusters are grouped together if they express similar top marker genes and are in close contact with each other on the UMAP plot. The marker gene sets are summarized in Table S4.

3) The above post-clustering Seurat object was exported to the monocle R package (v2.16.0) for pseudotime analysis (Qiu et al., 2017). For cell ordering, we used the DEGs derived from the above bulk RNA-seq experiments instead of the highly expressed and highly variable genes computed from the scRNA-seq datasets, out of the concern that the latter was enriched with highly expressed house keeping genes but not developmentally important transcription factors that are generally expressed at low levels. After estimating the size factors and dispersions of the dataset, reduceDimension (max_components=2, method='DDRTree') was performed to build the trajectory tree. The state containing the largest fraction of cells in the control sample was designated as the root state. Branch analysis was performed using the BEAM function. Psuedotime-dependent genes were identified using differentialGeneTest(fullModelFormulaStr="~sm.ns(Pseudotime)"). Significant genes were those with qvalue < 1e-3. These results are summarized in Table S5.

4) SCENIC analysis was conducted using the pySCENIC workflow (version 0.9.18) (Van de Sande et al., 2020). Gene-motif ranking using 10 kb around the transcription start site (TSS) were used to determine the search space around the TSS, and a 20-thousand motif database was used for RcisTarget and GRNBoost2 to identify co-expression modules and regulons. The scores of the activity of each regulon were computed by AUCell (Van de Sande et al., 2020). Each cell's clusterID was imported from pre-processed Seurat objects. Regulon specificity

scores were the means of regulon activity scores of cells in each cluster. These results are summarized in Table S6.

ATAC-seq

ATAC-seq was performed using 50,000 cells according to the fast ATAC-seq protocol (Corces et al., 2017). The resulted libraries were mixed and sequenced by Illumina Xten at the depth of 40-60 million 150 bp paired-end reads per sample. De-mutiplexed reads were mapped to the human genome (hg19) using Bowtie2 (v2.2.9) with default settings(Langmead and Salzberg, 2012). PCR duplicates were removed by SAMtools (v1.3.1) (Li et al., 2009). ATAC-seq peaks were called by MACS2 (v2.1.1.20160309) (Liu, 2014) using the following parameters: -f BAMPE -g hg -B -q 0.01. After removing ENCODE blacklisted regions(Amemiya et al., 2019), reproducible peaks between experimental repeats were used to assemble a merged peak set from all experiments. This merged peak set was used as input to generate a sequencing reads count table using the multicov function of bedtools (v2.25.0) (Quinlan and Hall, 2010). The count table was imported to the TCseq R package (v1.8.0) for time series analysis to identify peaks showing time-dependent changes of accessibility. Heatmaps of ATAC-seq peaks were generated by deepTools (v2.4.2) (Ramirez et al., 2016). Gene ontology enrichment analysis of the ATAC-seq experiments was performed using GREAT (v4.0.4) (McLean et al., 2010). Enriched TF binding motifs in each peak set were analyzed using the findMotifsGenome.pl function of HOMER (v4.1) (Heinz et al., 2010). The dynamically changed ATAC-seq peak sets are summarized in Table S7.

References

Amemiya, H.M., Kundaje, A., and Boyle, A.P. (2019). The ENCODE Blacklist: Identification of Problematic Regions of the Genome. Scientific reports *9*, 9354.
Bray, N.L., Pimentel, H., Melsted, P., and Pachter, L. (2016). Near-optimal probabilistic RNA-seq quantification. Nature biotechnology *34*, 525-527.
Butler, A., Hoffman, P., Smibert, P., Papalexi, E., and Satija, R. (2018). Integrating single-cell transcriptomic data across different conditions, technologies, and species. Nature biotechnology *36*, 411-420.

Corces, M.R., Trevino, A.E., Hamilton, E.G., Greenside, P.G., Sinnott-Armstrong, N.A., Vesuna, S., Satpathy, A.T., Rubin, A.J., Montine, K.S., Wu, B., *et al.* (2017). An improved ATAC-seq protocol reduces background and enables interrogation of frozen tissues. Nature methods *14*, 959-962.
Heinz, S., Benner, C., Spann, N., Bertolino, E., Lin, Y.C., Laslo, P., Cheng, J.X., Murre, C., Singh, H., and Glass, C.K. (2010). Simple combinations of lineage-determining transcription factors prime cis-regulatory elements required for macrophage and B cell identities. Molecular cell *38*, 576-589.
Langmead, B., and Salzberg, S.L. (2012). Fast gapped-read alignment with Bowtie 2. Nature methods *9*, 357-359.
Lanotte, M., Martin-Thouvenin, V., Najman, S., Balerini, P., Valensi, F., and Berger, R. (1991). NB4, a maturation inducible cell line with t(15;17) marker isolated from a human acute promyelocytic leukemia (M3). Blood *77*, 1080-1086.
Li, H., Handsaker, B., Wysoker, A., Fennell, T., Ruan, J., Homer, N., Marth, G., Abecasis, G., Durbin, R., and Genome Project Data Processing, S. (2009). The Sequence Alignment/Map format and SAMtools. Bioinformatics *25*, 2078-2079.
Liu, T. (2014). Use model-based Analysis of ChIP-Seq (MACS) to analyze short reads generated by sequencing protein-DNA interactions in embryonic stem cells. Methods in molecular biology *1150*, 81-95.
Love, M.I., Huber, W., and Anders, S. (2014). Moderated estimation of fold change and dispersion for RNA-seq data with DESeq2. Genome biology *15*, 550.
Macosko, E.Z., Basu, A., Satija, R., Nemesh, J., Shekhar, K., Goldman, M., Tirosh, I., Bialas, A.R., Kamitaki, N., Martersteck, E.M., *et al.* (2015). Highly Parallel Genome-wide Expression Profiling of Individual Cells Using Nanoliter Droplets. Cell *161*, 1202-1214.
McLean, C.Y., Bristor, D., Hiller, M., Clarke, S.L., Schaar, B.T., Lowe, C.B., Wenger, A.M., and Bejerano, G. (2010). GREAT improves functional interpretation of cis-regulatory regions. Nature biotechnology *28*, 495-501.
Newman, A.M., Liu, C.L., Green, M.R., Gentles, A.J., Feng, W., Xu, Y., Hoang, C.D., Diehn, M., and Alizadeh, A.A. (2015). Robust enumeration of cell subsets from tissue expression profiles. Nature methods *12*, 453-457.
Qiu, X., Mao, Q., Tang, Y., Wang, L., Chawla, R., Pliner, H.A., and Trapnell, C. (2017). Reversed graph embedding resolves complex single-cell trajectories. Nature methods *14*, 979-982.
Quinlan, A.R., and Hall, I.M. (2010). BEDTools: a flexible suite of utilities for comparing genomic features. Bioinformatics *26*, 841-842.
Ramirez, F., Ryan, D.P., Gruning, B., Bhardwaj, V., Kilpert, F., Richter, A.S., Heyne, S., Dundar, F., and Manke, T. (2016). deepTools2: a next generation web server for deep-sequencing data analysis. Nucleic acids research *44*, W160-165.
Van de Sande, B., Flerin, C., Davie, K., De Waegeneer, M., Hulselmans, G., Aibar, S., Seurinck, R., Saelens, W., Cannoodt, R., Rouchon, Q., *et al.* (2020). A scalable SCENIC workflow for single-cell gene regulatory network analysis. Nature protocols *15*, 2247-2276.

Supplemental Figures

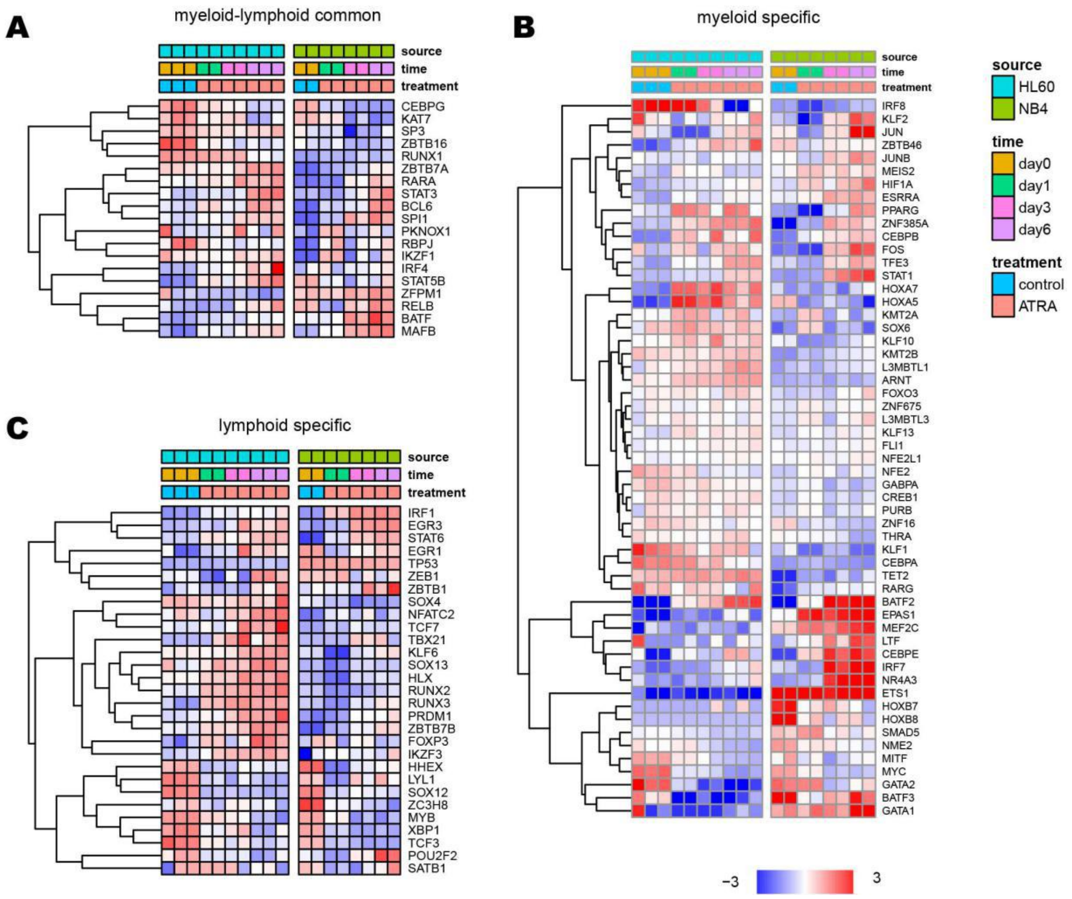

Figure S1. RNA-seq of TFs involved in myeloid and lymphocyte differentiation. Related to Figure 1.

Heatmap of RNA-seq data of genes associated with genes associated with both myeloid- and lymphoid-differentiation (A), with myeloid-differentiation only (B), or with lymphoid-differentiation only (C).

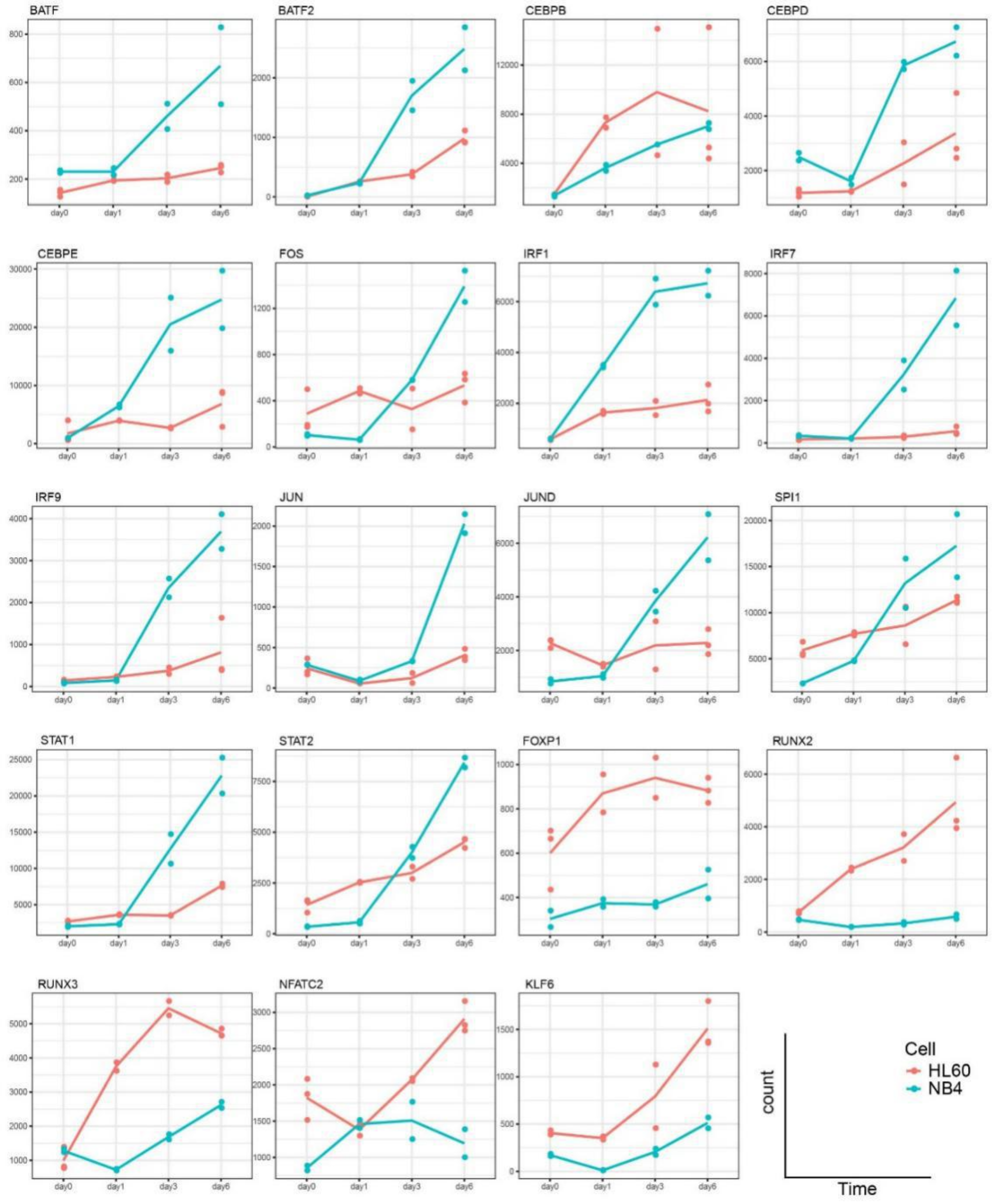

Figure S2. Expression of TFs regulating myeloid and lymphoid differentiation. Related to Figure 1.

RNA-seq data of in HL60 (red) and NB4 (blue) cells treated with 1 μM ATRA for 0/1/3/6 days. Y-axis shows RNA-seq counts of each each gene. X-axis shows the time.

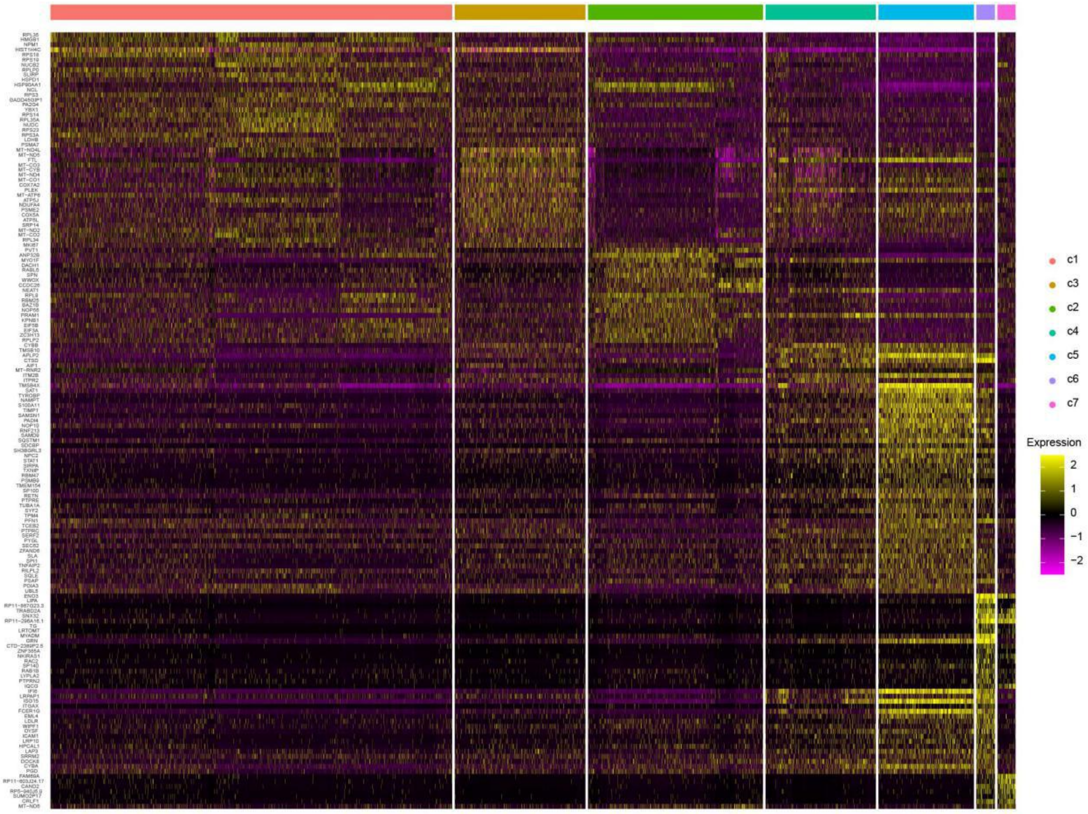

Figure S3. NB4 scRNA-seq analysis. Related to Figure 2.

Heatmap of cluster marker genes. Genes names are shown on the left. Cluster IDs are shown in the right.

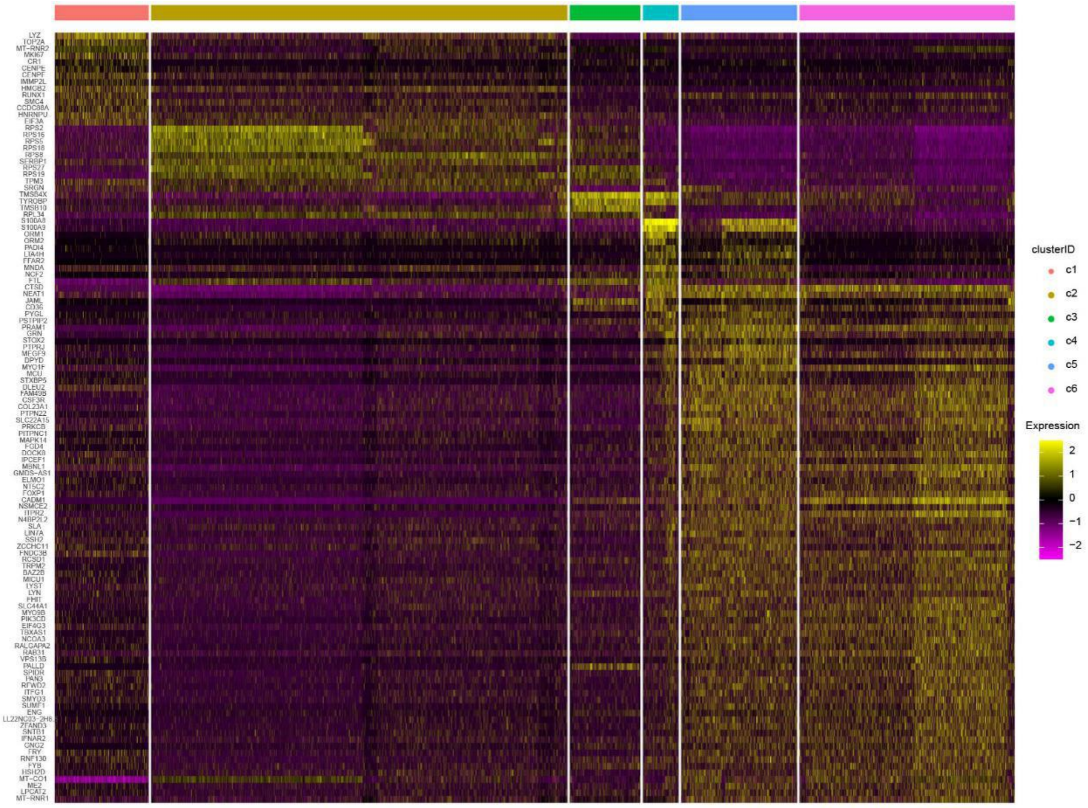

Figure S4. HL60 scRNA-seq analysis. Related to Figure 3.

Heatmap of cluster marker genes. Genes names are shown on the left. Cluster IDs are shown in the right.

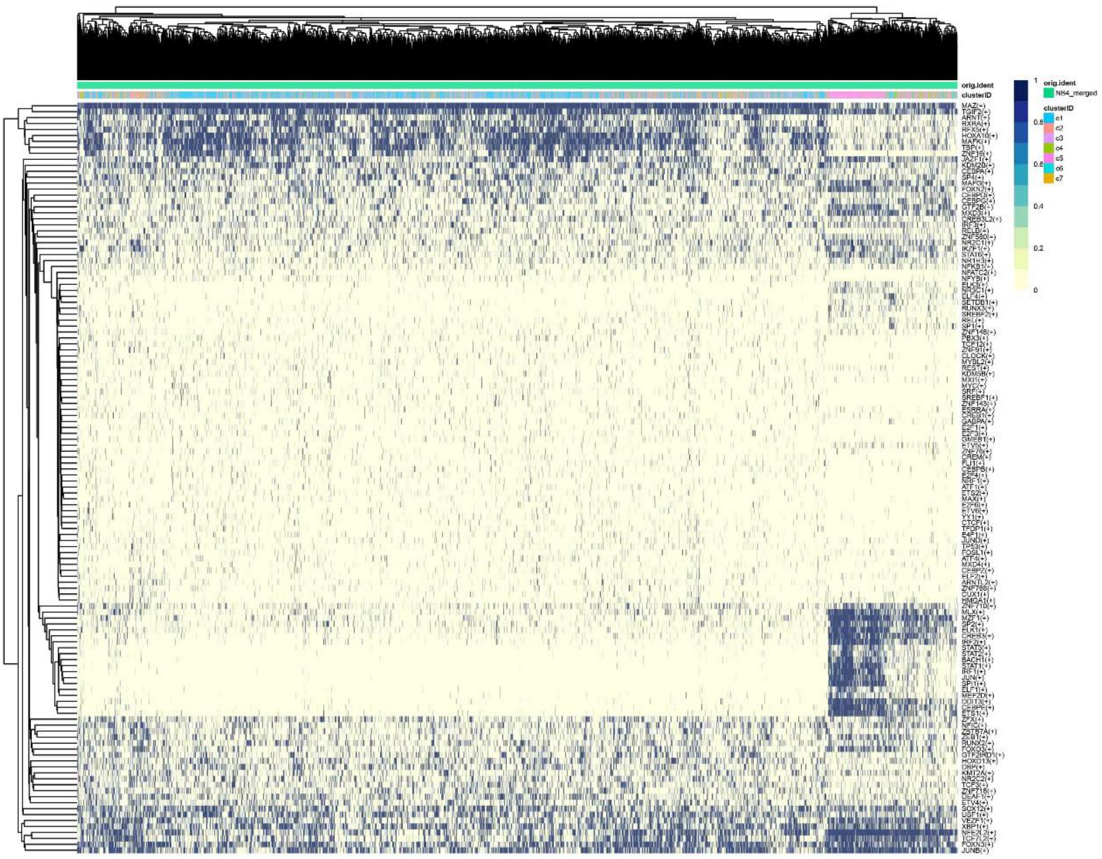

Figure S5. SCENIC analysis of NB4 scRNA-seq data. Related to Figure 6.

Heatmap binarized regulon activity scores of NB4. The names of TFs of each regulon are shown on the right.

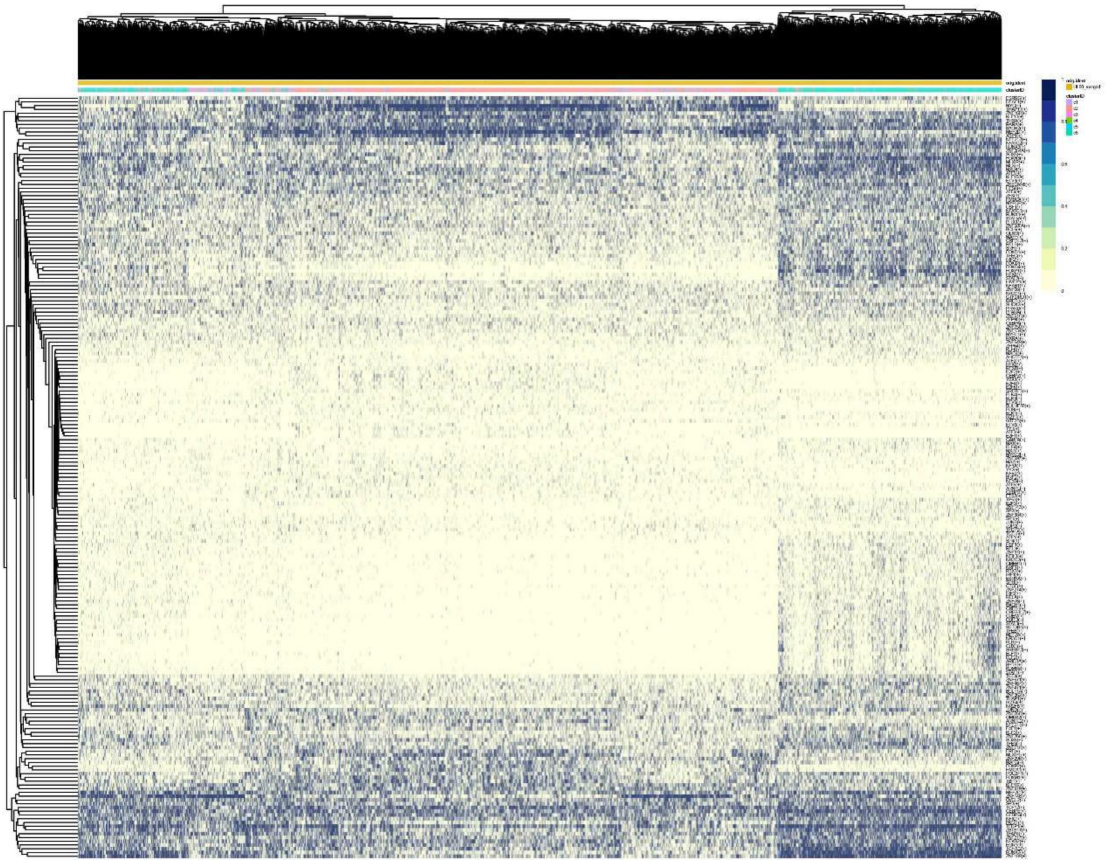

Figure S6. SCENIC analysis of HL60 scRNA-seq data. Related to Figure 6.

Heatmap binarized regulon activity scores of NB4. The names of TFs of each regulon are shown on the right.
